# Testing for equality of distributions using the concept of (niche) overlap

**DOI:** 10.1007/s00362-021-01239-y

**Published:** 2021-05-26

**Authors:** Judith H. Parkinson-Schwarz, Arne C. Bathke

**Affiliations:** Department of Mathematics, University of Salzburg, Hellbrunner Straße 34, 5020 Salzburg, Austria

**Keywords:** Equality test, Non-parametric, Overlap, Rank statistic, Relative effect, 62G10, 62G05

## Abstract

In this paper, we propose a new non-parametric test for equality of distributions. The test is based on the recently introduced measure of (niche) overlap and its rank-based estimator. As the estimator makes only one basic assumption on the underlying distribution, namely continuity, the test is universal applicable in contrast to many tests that are restricted to only specific scenarios. By construction, the new test is capable of detecting differences in location and scale. It thus complements the large class of rank-based tests that are constructed based on the non-parametric relative effect. In simulations this new test procedure obtained higher power and lower type I error compared to two common tests in several settings. The new procedure shows overall good performance. Together with its simplicity, this test can be used broadly.

**Supplementary Information:**

The online version contains supplementary material available at 10.1007/s00362-021-01239-y.

## Introduction

Analyzing data sets appropriately is of immense importance in any discipline. Not only are the numerical characteristics of the individual data sets of interest, but often their distribution in comparison to other data sets. In production one would like to know which process is more efficient or which product has higher quality, or in ecology one is interested in the overlap of the survival space of two species, just to name some examples.

For univariate data many tests have been proposed for the comparison of distributions prominently including those by Kolmogorov (1933) and Smirnov (1939), and the Cramér–von Mises test. The two-sample rank sum test due to Wilcoxon (1945) and Mann and Whitney (1947) assesses whether observations of one distribution tend to larger values than those from the other. The elementary concepts of these tests are likely key to their success, as even without a strong mathematical background it is possible to grasp the main concept behind the statistics. Of course, these classical tests also have their shortcomings which has sparked plenty of adaptations in order to enhance their performance. However, the adaptations have often complicated the tests too much to get well established themselves, or they are only useful in rather special situations. Consider, for example Khamis (1990), Drezner et al. (2010), or Baringhaus and Kolbe (2015). Other tests only focus on location or scale differences, see for example Marozzi (2012). More recent tests for equality of distributions like for example Ping (2000), Bera et al. (2013), and Wan et al. (2018) have been proposed but haven’t been able to establish themselves.

In clinical studies resources of participants or patients are often limited, due to ethical reasons, limited budget, or other reasons, leading to small sample sizes. With only a small sample it regularly is difficult to assess whether all model assumptions of a test are met and the results are reliable. It is thus of interest to have tests available with as little requirements as possible and yet good performances, i.e. reliable results. Likewise, plenty of literature show that non-parametric or quantile-based methods are generally appealing for new test methods, compare for example Al-Mutairi and Stat Papers (2017), Hassler (2018), Soni et al. (2019), Zamanzade (2019), or Jokiel-Rokita and Topolnicki (2019).

In this paper, we propose a new and easily motivated non-parametric test with competitive performance and straightforward interpretation. Based on the non-parametric relative effect, a quantity that has received renewed attention lately, due to its favorable properties, see Brunner et al. (2018) or Dastbaravarde and Zamanzade (2017), the test concentrates on an easy interpretation. Using the approach of Parkinson et al. (2018), we propose a fully non-parametric testing method that can easily be performed using ranks. The original intent of the cited paper was to measure overlap of two distributions representing ecological niches. The overlap can be considered a measure of similarity between two species. As those results are not only applicable to the quantification of niches but to any arbitrary data set, this measure of overlap is an adequate basis for a test statistic for testing whether two data sets are drawn from the same distribution or not. Instead of considering only a certain location parameter, such as mean or median, the niche overlap measure takes the full distributions into consideration. This results in a test for equality of distributions which is based on the full set of quantiles.

In Sect. 2 we present the necessary results of Parkinson et al. (2018) and describe the test procedure. Intensive simulations on the performance of the new testing method, also in comparison to other tests, are presented in Sect. 3 followed by a short conclusion and discussion in Sect. 4.

## Mathematical background and theoretical results

In the following we propose a test for equality of distributions based on the niche overlap value as defined and estimated by Parkinson et al. (2018). The notation in this paper is identical to theirs. We will first state the relevant results of that paper before introducing the new testing method.

Consider two groups of observations $X_1, \ldots , X_n$ and $Y_1, \ldots , Y_m$. We will assume that $X_1, \ldots , X_n$ are independent, identically distributed samples drawn from a continuous distribution function *F*, while $Y_1, \ldots , Y_m$ are independent, identically distributed samples drawn from a continuous distribution function *G*. The empirical estimators of *F* and *G* are denoted by $\hat{F}_n$ and $\hat{G}_m$, respectively.

In order to quantify how *F* is “contained” within *G* the statistical functional

$$\begin{aligned} I_2 = 2 \Big [ \int _{F^{-1}(1/2)}^\infty G(t) dF(t) - \int _{- \infty }^{F^{-1}(1/2)} G(t) dF(t) \Big ], \end{aligned}$$

was considered, as well as $I_1$, with the roles of *F* and *G* switched.

To explain how $I_1$ and $I_2$ quantify the inclusion of *F* in *G* and vice versa, consider four random variables $X^{(1)}$, $X^{(2)}$, $Y^{(1)}$, and $Y^{(2)}$ which can be constructed from *X* and *Y*. Denote the distribution of *F* below its median as $F_1$ and above its median as $F_2$, such that

1 $$\begin{aligned} F_1(t)&=\left\{ \begin{array}{ll}2 F(t), &{} t< F^{-1}(1/2),\\ 1, &{} t \ge F^{-1}(1/2), \end{array} \right. ~ F_2(t)&=\left\{ \begin{array}{ll} 0, &{} t< F^{-1}(1/2),\\ 2 F(t)-1, &{} t \ge F^{-1}(1/2). \end{array} \right. \end{aligned}$$

Then one can construct the random variables $X^{(1)} \sim F_1$ and $X^{(2)} \sim F_2$ by conditioning on $X \le F^{-1}(0.5)$ and $X \ge F^{-1}(0.5)$, respectively, and similar for $Y^{(1)}$ and $Y^{(2)}$. Now $I_2 = P(X^{(1)} \le Y \le X^{(2)})$ and $I_1 = P(Y^{(1)} \le X \le Y^{(2)})$ holds for proof we refer to Parkinson et al. (2018).

The following important properties hold for $I_1$ and $I_2$. As $I_1$ and $I_2$ can be interpreted as probabilities it holds that $I_1+I_2 \in [0,1]$ for *F* and *G* absolutely continuous. If *G* is continuous and $F=G$ then $I_1=I_2=1/2$. Additionally, $I_1$ and $I_2$ are invariant under strictly monotone, continuous transformations, where the same transformation $\phi$ is being applied to both *F* and *G*. For further properties and the proofs of the stated properties please consult Lemma 2.2 of Parkinson et al. (2018).

### Lemma 1

(Lemma 2.19, Parkinson et al. 2018) Let *F* and *G* be continuous distribution functions and $F^{-1}(1/2) = G^{-1}(1/2)$ then $I_1 +I_2 =1$.

In order to construct an estimator for $I_2$, all observations of both groups shall be ranked. Without loss of generality we will assume that $X_1< X_2< \cdots < X_n$ (to simplify notation). All the *X*-observations below their median are $X_1, \ldots , X_K$ where *K* is the largest integer with $K \le (n+1)/2$. Their ranks within both groups will be denoted by $R^{X<}_1,\ldots ,R^{X<}_K$, the remaining ranks by $R^{X>}_{K+1},\ldots ,R^{X>}_n$. In case of ties we will use midranks. Further define

$$\begin{aligned} R^{X<}_\cdot = \sum _{i=1}^K R^{X<}_i \quad \text { and } \quad R^{X>}_\cdot = \sum _{i=K+1}^n R^{X>}_i. \end{aligned}$$

### Lemma 2

(Lemma 2.11, Parkinson et al. 2018) For $R^{X>}_\cdot$ and $R^{X<}_\cdot$ defined as above, a consistent estimator for $I_2$ is given by

$$\begin{aligned} \hat{I}_2 = \frac{2}{m n} (R^{X>}_\cdot - R^{X<}_\cdot ) + \frac{1}{2} c , \end{aligned}$$

with $c = - n/m$ for *n* even and $c \approx - n/m$ for *n* odd, and *n* and *m* large. A similar consistent estimator for $I_1$ can be provided.

### Theorem 1

Let *F* and *G* be continuous distribution functions with $F^{-1}(1/2) =G^{-1}(1/2)$. Then the estimators of $I_1$ and $I_2$ as given in Lemma 2 are biased. More precisely, $\mathbb {E} [ \hat{I}_1 + \hat{I}_2 ] < 1$.

### Proof

As stated in Lemma 1$I_1+I_2 =1$ if $F^{-1}(1/2) =G^{-1}(1/2)$. To show that the estimators are biased we will show that the expectation of the sum of the two estimators is systematically below one, thus implying that at least one of the two estimators must be biased. From the fact that $I_1$ and $I_2$ are symmetric it should be self-evident that the bias arises from the combination of the two estimators.

Without loss of generality, assume $X_1< X_2< \cdots < X_n$ and $Y_1< Y_2< \cdots < Y_m$ with *K* and *L* denoting the indices of the respective medians of the *X* and the *Y* samples. Due to the fact that the underlying distributions are continuous we do not consider the case of ties between the observations as the probability for this is zero.

We will consider the expression of the estimators through indicator functions, i.e.

$$\begin{aligned} \hat{I}_1 + \hat{I}_2&= \frac{2}{mn} \Bigg [\Bigg (\sum \limits _{j=L+1}^m \! \sum \limits _{i=1}^n \! \mathbb {1}\{ X_i< Y_j \} - \sum \limits _{j=1}^L \! \sum \limits _{i=1}^n \! \mathbb {1}\{ X_i< Y_j \}\Bigg ) \\&\quad \times \Bigg (\sum \limits _{i=K+1}^n \! \sum \limits _{j=1}^m \! \mathbb {1}\{ Y_j< X_i \} - \sum \limits _{i=1}^K \! \sum \limits _{j=1}^m \! \mathbb {1}\{ Y_j < X_i \} \Bigg ) \Bigg ]. \end{aligned}$$

Rearranging the expression we obtain

$$\begin{aligned} \frac{2}{mn} \Big [&\sum \limits _{i=1}^K \! \sum \limits _{j=L+1}^m \! ( \mathbb {1}\{X_i< Y_j\} - \mathbb {1}\{ Y_j< X_i \} ) + \sum \limits _{i=K+1}^n \! \sum \limits _{j=1}^L \! ( \mathbb {1}\{ Y_j< X_i \} - \mathbb {1} \{X_i< Y_j\} ) \\&\sum \limits _{i=1}^K \! \sum \limits _{j=1}^L \! (- \mathbb {1}\{Y_j< X_i \} - \mathbb {1}\{ X_i< Y_j \} ) + \sum \limits _{i=K+1}^n \! \sum \limits _{j=L+1}^m \! ( \mathbb {1}\{ X_i< Y_j\} + \mathbb {1}\{ Y_j < X_i \} ) \Big ] ~ . \end{aligned}$$

Now we will consider the two lines separately, ignoring the factor 2/*mn* for the time being.

In the second line the indicators are complimentary, respectively, as $- \mathbb {1}\{Y_j< X_i \} - \mathbb {1}\{ X_i < Y_j \} = -1$ and $\mathbb {1}\{ X_i< Y_j\} + \mathbb {1}\{ Y_j < X_i \} =1$. This means that the second line can be reduced to

2 $$\begin{aligned} K \cdot L \cdot (-1) + (n -(K+1))(m- (L+1)) \cdot 1 \end{aligned}$$

As for the first line, we will immediately consider its expectation. Rearranged we obtain

3 $$\begin{aligned} \sum \limits _{i=1}^K \! \sum \limits _{j=L+1}^m \! (2 \mathbb {E}[ \mathbb {1}\{X_i< Y_j\}] - 1 ) + \sum \limits _{i=K+1}^n \! \sum \limits _{j=1}^L \! (1- 2 \mathbb {E}[ \mathbb {1}\{ X_i < Y_j \}] ). \end{aligned}$$

As we are showing that the expectation of the sum of the estimators remains systematically below 1, we will simply show that even if (3) is maximized it is smaller than 1. The indicator function can only take the values 0 and 1 such that the expectation of it lies in the interval [0, 1]. Now (3) will be maximized if the first expectation takes the value 1 and the second expectation takes value 0. Then the maximization of (3) is given through

4 $$\begin{aligned} K \cdot (m-(L+1)) + (n-(K+1)) \cdot L ~. \end{aligned}$$

Taking (2) and (4) we obtain

$$\begin{aligned} \mathbb {E} [ \hat{I}_1 + \hat{I}_2 ]&\le \frac{2}{mn} \Big [ K \cdot (m-L-1) + L \cdot (n-K-1) -KL +(n-K-1)(m-L-1) \Big ] \\&= \frac{2}{mn} \Big [ mn - 2LK - m-n-1 \Big ] ~. \end{aligned}$$

To further simplify that term, we will assume that *m* and *n* are even such that $m=2L$ and $n=2K$. Then we have

$$\begin{aligned} \mathbb {E} [\hat{I}_1 + \hat{I}_2]&\le \frac{2}{2L2K} \Big [2L2K -2LK-2L-2K+1 \Big ] \\&= \frac{1}{2LK} \Big [ 2LK - (2L+2K-1) \Big ] \\&= 1 - \frac{2L+2K-1}{2LK} < 1, \end{aligned}$$

as the term $(2L+2K-1)/2LK$ is positive for all $L, K \in \mathbb {N}$.

Similar, it can be shown that $\mathbb {E}[ \hat{I}_1 + \hat{I}_2 ]$ is truly smaller than 1, if *n*, *m*, or both are uneven. Thus we can conclude that $\hat{I}_1+\hat{I}_2$ is a biased estimator for $I_1+I_2$ if $F^{-1}(1/2)=G^{-1}(1/2)$. $\square$

### Remark 1

The bias of $\hat{I}_1 + \hat{I}_2$ depends on the two probabilities $P(X_K<Y_{L+1})$ and $P(Y_L< X_{K+1})$. The larger those two are the smaller the bias will be.

### Lemma 3

Under certain criteria that are specified in detail in Parkinson et al. (2018), $(n+m)^{1/2} \left( \hat{I}_2 - I_2 \right)$ converges to a normal distribution with expectation zero.

Based on the above Lemmas, $I_2$ can be consistently estimated, and asymptotic inference for $I_2$ will be possible based on the rank statistics, provided the variance of $(n+m)^{1/2} \left( \hat{I}_2 - I_2 \right)$ can also be consistently estimated. For a special case, we can provide a consistent rank-based variance estimator

In the following assume $G^{-1}(0.5) = F^{-1}(0.5)$ holds, that is, the distributions *F* and *G* are assumed to have the same median. In this case, $I_2$ can be expressed through four random variables that can be constructed from *X* and *Y*. This result, along with an approach introduced by Konietschke (2009), provides the following way to construct a variance estimator.

Analogously to the *X*-sample earlier, without loss of generality, assume that $Y_1< Y_2< \cdots < Y_m$ and divide them into two groups at the index *L*, with *L* the largest integer for which $L \le (m+1)/2$. Additionally, without loss of generality, split the sample of *X* at the index *K* such that $X_1,\ldots ,X_K$ are below and $X_{K+1},\ldots ,X_n$ are above the median. We then compare the samples of *X* and *Y* which are below their respective medians with each other. We denote their ranks in the combined group by $R_i^{X(<)}$ and $R_i^{Y(<)}$ for $X_1, \ldots , X_K$ and $Y_1, \ldots , Y_L$ and their averages by $\bar{R}_\cdot ^{X(<)}$ and $\bar{R}_\cdot ^{Y(<)}$. Similarly define $\bar{R}_\cdot ^{X(>)}$ and $\bar{R}_\cdot ^{Y(>)}$ for those that are above their respective medians. Finally, the observations are (additionally) ranked only within each of these four groups and these within-group ranks are denoted by $R_i^{(X<)}$, $R_i^{(Y<)}$, $R_i^{(X>)}$, and $R_i^{(Y>)}$, respectively.

### Lemma 4

(Lemma 2.20, Parkinson et al. 2018) Let *F* and *G* be continuous distribution functions. Assume $G^{-1}(1/2) =F^{-1}(1/2)$ holds true. Further, define

$$\begin{aligned} s_{X1}^2= & {} \frac{1}{L^2 (K-1)} \; \sum \limits _{i=1}^K \! \big ( R_i^{X(<)} - R_i^{(X<)} - \bar{R}_\cdot ^{X(<)} + \frac{K+1}{2} \big )^2 \; ,\\ s_{X2}^2= & {} \frac{1}{(m-L)^2 (n-K-1)} \; \sum \limits _{j=K+1}^{n} \! \big ( R_j^{X(>)} - R_j^{(X>)} - \bar{R}_\cdot ^{X(>)} + \frac{n-K+1}{2} \big )^2 \; , \end{aligned}$$

and $s_{Y1}^2$ and $s_{Y2}^2$ analogously. A consistent variance estimator for $\hat{I}_2$ is

$$\begin{aligned} s_2^2=\frac{L+K}{2} \big ( \frac{s_{X1}^2}{K} + \frac{s_{Y1}^2}{L} \big ) + \frac{n+m-L-K}{2} \big ( \frac{s_{X2}^2}{n-K} + \frac{s_{Y2}^2}{m-L} \big ) \; . \end{aligned}$$

Here, consistency is to be understood as $Var(\hat{I}_2)/s_2^2 \rightarrow 1$ in probability.

This is the same variance estimator as for $\hat{I}_1$.

### Theorem 2

Let $I_1$ and $I_2$ be defined as before, and $\hat{I}_1$ and $\hat{I}_2$ denote the rank estimators as introduced above. Further let $s_2^2$ be the variance estimator as given in Lemma 4. Consider the null hypothesis $H_0 : F \equiv G$ versus $H_1 : F \ne G$ and the test statistics $NO_i$, $i=1,2$, defined by

$$\begin{aligned} NO_i := \sqrt{n+m} \left( \frac{\hat{I}_i -0.5}{s_2} \right) \;. \end{aligned}$$

Under the null hypothesis $NO_i$, $i=1,2$ is asymptotically distributed according to a standard normal distribution.

### Remark 2

Instead of the standard normal distribution, one may also approximately use a t-distribution with $n+m-1$ degrees of freedom. Simulations showed that, especially for small sample sizes, the *t*-distribution provided a better approximation to the sampling distribution of $NO_i$.

Using the results of Theorem 2 we can design a test for equal distributions.

### Theorem 3

Consider the null hypothesis $H_0 : F \equiv G$ versus the alternative $H_1 : F \ne G$. Under the null hypothesis it holds that $NO_i$, $i=1,2$, is asymptotically distributed according to a standard normal procedure. Denote with $p_1$ and $p_2$ the, through the Bonferroni–Holm procedure, adjusted p-values of the test statistics $NO_1$ and $NO_2$, respectively. Then $H_0$ is to be rejected if $p_1<\alpha$ or $p_2 < \alpha$.

### Remark 3

Due to the way the variance estimator is constructed, it is possible that it can be zero. This can occur if the assumption of equal medians is so strongly violated in the data set that the true proportions of observations of $Y_1$ below the median of *X* and of $X_2$ above the median of *Y* or vice versa are (close to) zero. In these situations it intuitively appears justified to reject the null hypothesis.

In such cases only an upper p-value can be provided. Following the idea as given in Chapter 3.5.3 of Brunner et al. (2018), the data sets are shifted until the variance estimator is truly greater than zero. The resulting p-value of the test statistic based on the shifted data is an upper limit of the true p-value.

### Remark 4

As shown earlier we have a bias for $\hat{I}_1$ and $\hat{I}_2$ under the null hypothesis and all alternatives where the underlying distribution functions have equal medians. To minimize this bias we propose a modification to the existing procedure. When calculating $\hat{I}_2$ we will add an extra observation to the data set *Y*, namely the median of the *X*-observations. And vice versa for $I_1$. With this modification it is possible to obtain $I_1+I_2>1$ which reduces the bias of the estimators.

## Simulation

### General settings

In order to investigate the small sample properties of the test statistics concerning Type I and Type II error, we have performed simulation studies in R (R version 3.2.3, R Core Team, 2017). Additionally, simulations to check the correctness and robustness of the new test procedure were run. As to put the error rates into context, the Kolmogorov–Smirnov test and the Wilcoxon-rank-sum test were also calculated using the same data. All simulations for our testing method used the adaptation as proposed in Remark 4. Further simulations without the adaptation, which are omitted here , showed an inflated Type I error for small sample sizes.

### Empirical confirmation of Theorem 2

In this part we will confirm the limiting distribution as given in Theorem 2. Consider data distributed with $F=G=exp(0.5)$. The sample sizes in all 4 settings are equal and given by 20, 35, 50, 100. For each setting we ran 1000 simulations.

**Fig. 1:**
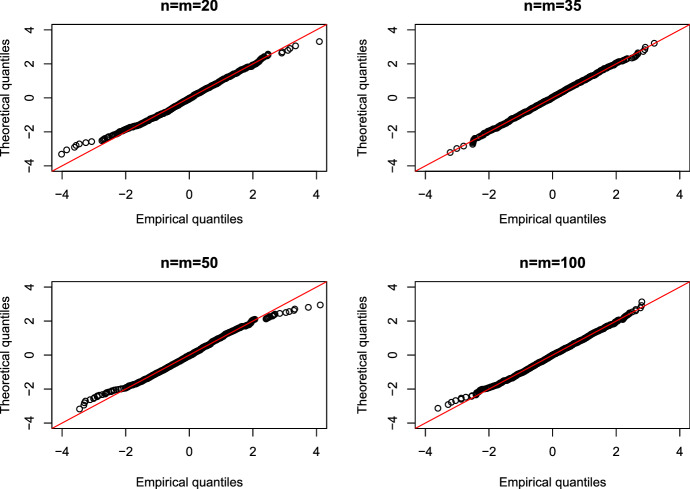
Q–Q plot of the empirical quantiles of $NO_1$ (x-axis) versus the theoretical $t_{n+m-1}$ percentiles (y-axis) for different sample sizes corresponding to the theoretical result in Theorem 2. The (red) line is the bisecting line. (Color figure online)

The Q–Q plots of the empirical quantiles of the test statistic $NO_1$ versus the *t*-distribution percentiles can be seen in Fig. 1. For all sample sizes the middle quantiles are adequate but bigger differences can be noticed for the outer quantiles. For higher sample sizes the empirical distribution agrees very well with the theoretically justified *t*-distribution. The corresponding Q–Q plots for $NO_2$ can be found in the supplementary material and show similar results.

### Visual power analysis

For the second set of simulations we analyses the power of the three tests, keeping one distribution fixed and varying the parameters of the second distribution. The sample sizes were $n=m=50$ in all settings. The results were then visualized for a first interpretation of the strengths of the individual tests. For all settings we ran 1000 simulations.

#### Setting 1

**Fig. 2:**
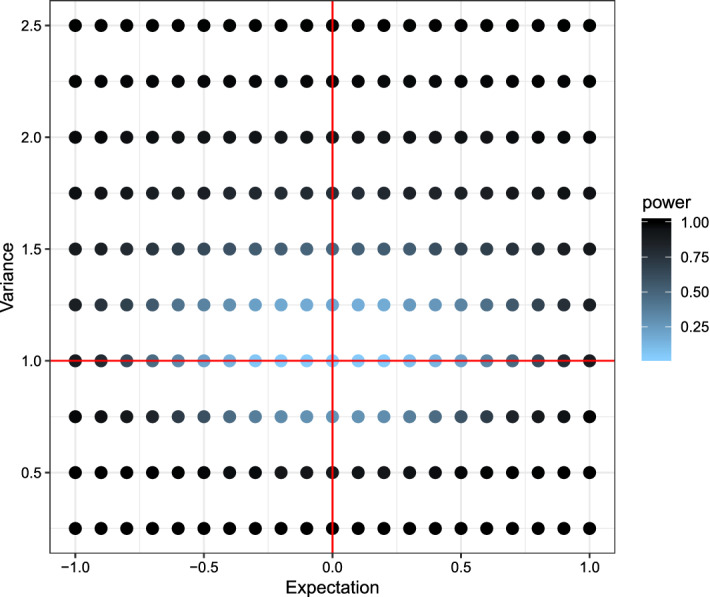
Plot of the power using the (niche) overlap test with respect to the expectation (x-axis) and variance (y-axis). Lighter colors indicate low power, darker colors high power. The red lines indicate the true expectation and variance of *X*, while expectation and variance for *Y* varies. (Color figure online)

**Fig. 3:**
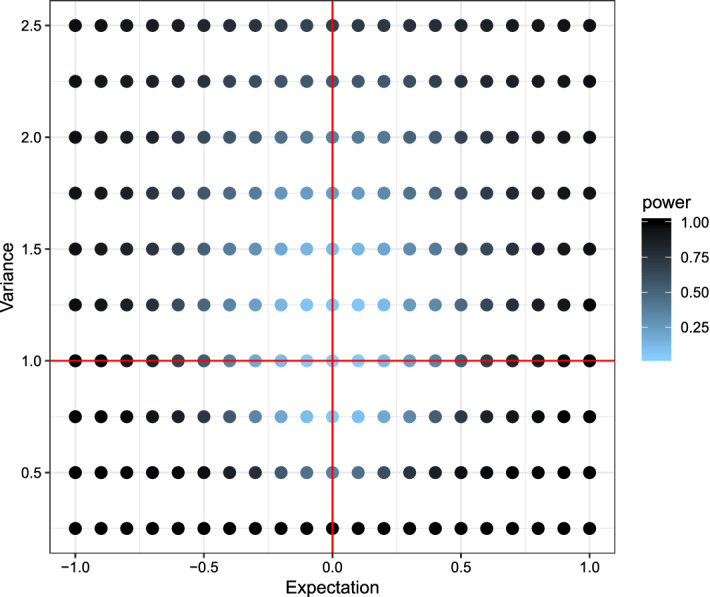
Plot of the power using the Kolmogorov–Smirnov test with respect to the expectation (x-axis) and variance (y-axis). Lighter colors indicate low power, darker colors high power. The (red) lines indicate the true expectation and variance of *X*, while expectation and variance for *Y* varies. (Color figure online)

**Fig. 4:**
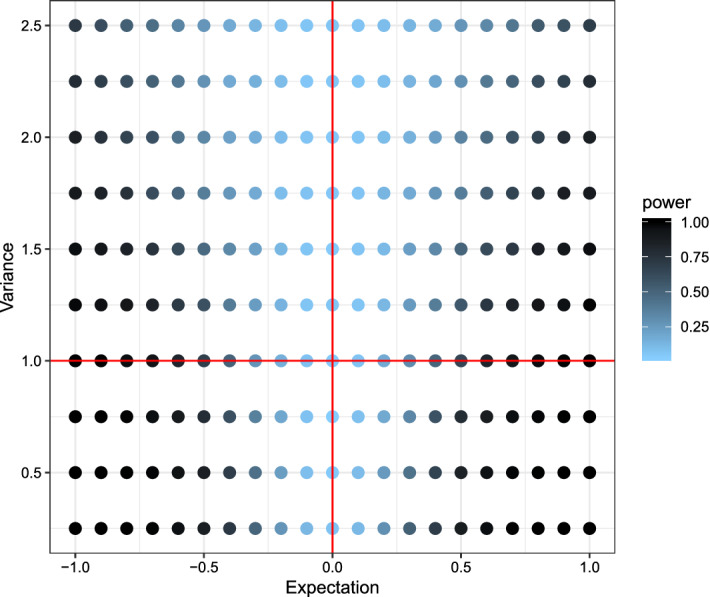
Plot of the power using the Wilcoxon-rank-sum test with respect to the expectation (x-axis) and variance (y-axis). Lighter colors indicate low power, darker colors high power. The (red) lines indicate the true expectation and variance of *X*, while expectation and variance for *Y* varies. (Color figure online)

In the first setting the *X*-observations were drawn from a normal distribution with mean 0 and variance 1. Several *Y*-observations were drawn also from a normal distribution but with different parameters ranging from $-1$ to 1 for the mean and 0.25 to 2.5 for the variance. The power for each combination was visualized in Fig. 2 for the new test, in Fig. 3 for the Kolmogorov–Smirnov test, and in Fig. 4 for the Wilcoxon-rank-sum test.

Comparing the three plots one can quite easily see some key-differences between the tests. While the newly proposed test had lower power for detecting small differences in the mean of the two distributions, it had the highest power out of the three for detecting small differences in the variances. The Kolmogorov–Smirnov test showed almost opposite behavior, uncovering small differences in the mean but not in the variance. Due to its reliance on the non-parametric relative effect the Wilcoxon-rank-sum test is only capable of detecting location differences in a location-scale model.

#### Setting 2

**Fig. 5:**
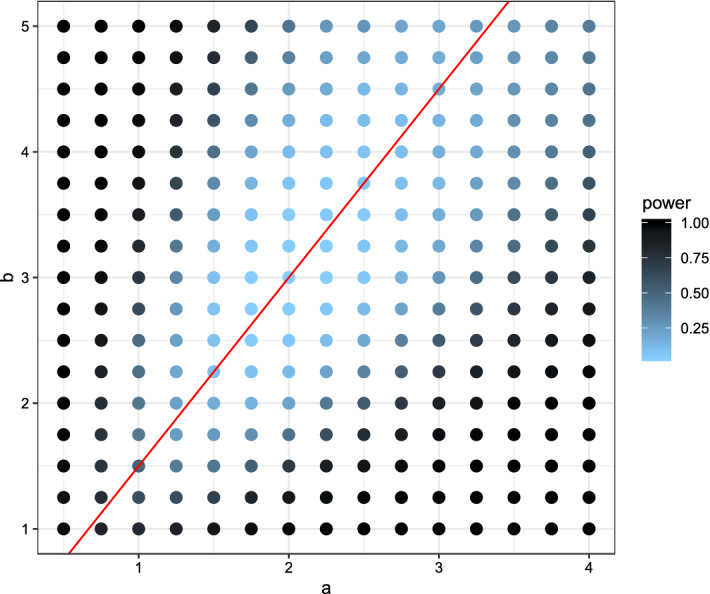
Plot of the power using the (niche) overlap test with respect to the parameters of the beta-distribution *a* on the x-axis and *b* on the y-axis. Lighter colors indicate low power, darker colors high power. The (red) line indicates the combinations of *a* and *b* for which the expectation is the same as for *X*. (Color figure online)

**Fig. 6:**
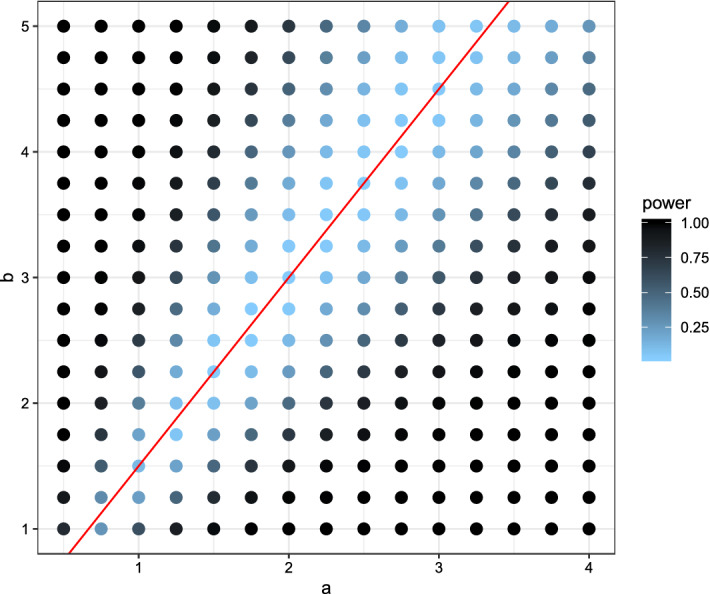
Plot of the power using the Kolmogorov–Smirnov test with respect to the parameters of the beta-distribution *a* on the x-axis and *b* on the y-axis. Lighter colors indicate low power, darker colors high power. The (red) line indicates the combinations of *a* and *b* for which the expectation is the same as for *X*. (Color figure online)

**Fig. 7:**
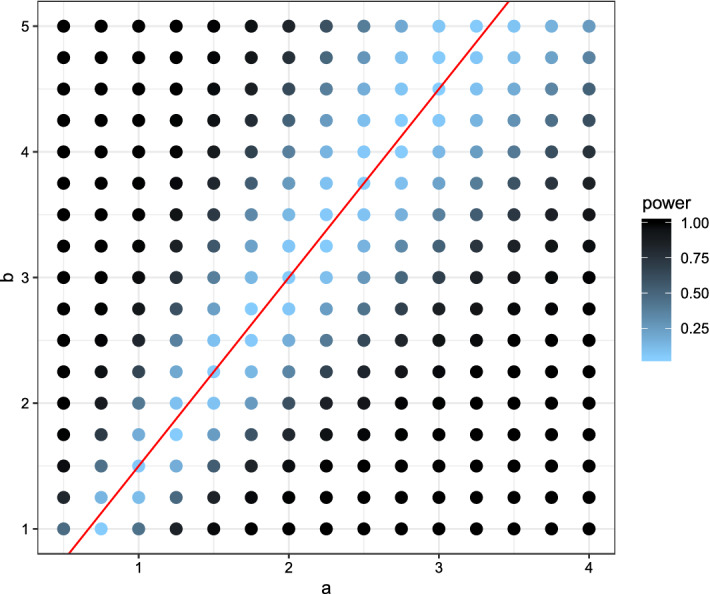
Plot of the power using the Wilcoxon-rank-sum test with respect to the parameters of the beta-distribution *a* on the x-axis and *b* on the y-axis. Lighter colors indicate low power, darker colors high power. The (red) line indicates the combinations of *a* and *b* for which the expectation is the same as for *X*. (Color figure online)

In the second setting the *X*-observations were drawn from a beta distribution with parameters $a=2$ and $b=3$. Several *Y*-observations were drawn from beta distributions with parameters ranging from 0.5 to 4 for *a* and 0.5 to 5.5 for *b*. The power for each combination was visualized in Fig. 5 for our test, in Fig. 6 for the Kolmogorov–Smirnov test, and in Fig. 7 for the Wilcoxon-rank-sum test.

In this settings both parameters take influence on the expectation and the variance. Along the red line all three tests only obtained a low power. The Kolmogorov–Smirnov test quickly increased the power when moving away from the scenario of equal expectations, see Fig. 6. Similar behavior could be observed when looking at the power plot of the Wilcoxon-rank-sum test, Fig. 7. Looking at Fig. 5 one notices bigger differences. Moving along the red line, and away from the true parameters, i.e. the scenario of equal expectations but differing variances, the newly introduced test increased its power faster than the other two tests. However moving away from the red line, the power increased more slowly.

### Error analysis based on sample size

In this part we calculated the Type I and Type II error for several different combinations of *F* and *G* and for different sample sizes. For all settings we ran 1000 simulations.

#### Type I error

In the following the observations were drawn from the same distribution $F=G$. We considered the distributions $F_1 = N(0,1)$, $F_2 = Exp(1/2)$, $F_3 = B(2,3)$, $F_4 = U(0,1)$, $F_5 = \mathscr {X}^2_1$, and $F_6 = t_{10}$ for sample sizes ranging from 10 to 100 in steps of 10. The sample sizes were equal in all settings.

**Table 1:** Type I error for several different distributions according to sample size for the (niche) overlap test (NO), the Kolmogorov–Smirnov test (KS), and the Wilcoxon-rank-sum test (WR)

	10	20	30	40	50	60	70	80	90	100
NO F1	5.5	3.0	3.1	2.8	3.2	3.4	2.9	3.0	3.4	3.4
NO F2	5.6	4.1	3.3	3.4	3.1	3.9	2.8	2.8	4.6	3.3
NO F3	5.4	5.1	4.0	3.9	3.1	3.8	3.9	3.4	3.4	3.4
NO F4	5.0	5.0	3.1	3.2	4.4	4.3	3.1	4.3	4.9	3.9
NO F5	4.6	2.8	4.1	3.5	4.0	4.0	3.7	3.9	3.2	3.7
NO F6	4.5	4.5	3.5	2.8	3.3	3.7	3.2	3.3	2.1	3.1
KS F1	1.2	3.0	4.0	3.6	4.1	5.3	2.6	4.0	3.1	4.2
KS F2	1.2	4.0	4.1	2.7	3.9	4.6	2.8	3.3	3.9	3.6
KS F3	1.1	3.4	3.8	3.8	4.1	4.2	3.3	3.7	4.4	4.0
KS F4	1.6	3.4	3.7	2.9	3.8	3.9	2.6	4.0	3.2	3.1
KS F5	0.7	3.1	2.4	2.5	3.4	6.0	3.3	3.6	3.7	3.3
KS F6	0.8	3.5	3.0	2.7	5.2	5.7	3.5	3.1	3.5	4.0
WR F1	4.8	4.6	6.6	5.6	4.8	5.7	3.9	5.6	4.3	5.2
WR F2	5.1	5.8	5.5	4.4	5.3	4.9	4.7	4.5	5.4	5.3
WR F3	3.9	5.7	5.3	5.8	5.9	3.9	5.4	4.7	6.3	5.0
WR F4	4.6	5.2	5.9	5.2	5.5	4.6	4.7	5.4	5.1	5.6
WR F5	3.9	4.9	3.4	5.1	4.1	4.8	5.5	4.2	4.2	4.6
WR F6	2.9	4.9	4.0	4.5	4.8	5.5	5.0	4.3	4.5	4.7

As one can see in Table 1 the new test obtained the nominal level of $\alpha =0.05$ in all settings for very small sample sizes ($n=m=10$, $n=m=20$), whereas for higher sample sizes, it was a little bit conservative. The Kolmogorov–Smirnov test was too conservative for small sample sizes. For moderate to higher sample sizes, the Kolmogorov–Smirnov test was closer to the nominal level but remained conservative. In the settings considered, the Wilcoxon-rank-sum test maintained the nominal level well for all sample sizes.

#### Type II error

The combination of distributions we analyzed can be found in Table 2. Again we considered sample sizes ranging from 10 to 100 in steps of 10. The sample sizes were equal in all settings.

**Table 2:** Different combinations of *F* and *G* as used to estimate the Type II error

Alternative	F	G	
A	*N*(0, 1)	*N*(0, 1.5)	Same expectation
B	*N*(0.5, 1)	*U*(0, 1)	Same expectation
C	*N*(0.4, 1)	*B*(2, 3)	Same expectation
D	*N*(0, 1)	*N*(0.5, 1)	Same variance
E	*N*(2, 1)	*Exp*(1)	Same variance
F	*N*(1, 1)	*Exp*(1)	Same expectation and variance
G	*N*(1, 2)	$\mathscr {X}^2_1$	Same expectation
			and variance

The simulation settings were chosen in a manner that the differences were hard to detect, using combinations of distributions with equal expectation, variance or both. This led to high Type II errors, especially for small sample sizes, but showed the differences between the tests more accurately. For bigger differences between the two distributions, all tests obtained low Type II errors and thus those simulations are omitted in this paper. The results of the simulations can be found in Table 3.

**Table 3:** Type II error for several different distributions according to sample size for the (niche) overlap test (NO), the Kolmogorov–Smirnov test (KS), and the Wilcoxon-rank-sum test (WR)

	10	20	30	40	50	60	70	80	90	100
NO A	90.1	81.5	69.4	57.2	47.0	42.4	33.4	26.3	23.3	16.7
NO B	49.1	8.8	1.5	0.4	0.0	0.0	0.0	0.0	0.0	0.0
NO C	22.6	1.3	0.1	0.0	0.0	0.0	0.0	0.0	0.0	0.0
NO D	87.4	86.4	82.5	80.2	77.6	76.2	72.6	73.2	69.1	64.7
NO E	49.4	22.3	9.7	4.4	2.4	0.3	0.3	0.1	0.0	0.0
NO F	93.1	89.9	88.3	87.2	84.8	80.1	80.7	75.9	72.0	69.6
NO G	73.6	49.3	31.4	19.7	11.4	5.1	2.7	2.1	0.7	0.5
KS A	98.2	94.7	92.2	91.2	89.1	83.3	86.8	84.3	81.4	76.8
KS B	93.0	62.4	29.3	11.3	1.6	0.1	0.0	0.0	0.0	0.0
KS C	88.4	38.4	8.8	1.5	0.0	0.0	0.0	0.0	0.0	0.0
KS D	93.6	80.7	70.6	61.9	47.7	37.2	36.0	27.9	20.2	15.9
KS E	60.2	10.1	2.6	0.3	0.0	0.0	0.0	0.0	0.0	0.0
KS F	97.7	90.9	86.4	86.2	79.9	67.6	74.1	60.5	51.3	48.4
KS G	94.6	73.5	49.0	33.1	10.9	2.3	1.5	0.3	0.1	0.0
WR A	95.6	94.4	94.7	94.4	95.8	94.7	95.1	96.3	95.9	95.8
WR B	92.6	93.4	92.7	92.8	93.2	92.9	92.0	92.8	93.6	91.2
WR C	92.3	92.0	91.9	90.8	91.7	92.0	91.1	92.1	93.7	91.0
WR D	83.4	70.1	52.7	43.5	33.4	25.7	17.4	13.3	8.8	7.1
WR E	37.7	7.7	1.7	0.2	0.1	0.0	0.0	0.0	0.0	0.0
WR F	95.0	92.1	91.4	91.1	90.4	88.1	89.6	86.4	84.3	84.3
WR G	92.2	92.7	91.5	91.9	92.1	93.1	91.8	90.1	89.1	90.2

Looking first at settings A, B, and C, where both groups had the same expectation we notice already great differences between the performances. In all three scenarios, as expected, the Wilcoxon-rank-sum test fails completely, even for sample sizes of $n=m=100$. The other two methods both were struggling with setting A where the distributions came from the same family, but improved with increased sample sizes. In the settings B and C both picked up the differences quite well, with low Type II error for sample sizes of $n=m=40$ and higher. In all three settings, the new (niche) overlap test however outperformed the Kolmogorov–Smirnov test.

In settings D and E the expectation differed but the variance was the same. Here the Wilcoxon-rank-sum test and the Kolmogorov–Smirnov test outperformed the new test. Even though the (niche) overlap test was able to pick up the differences, the required sample sizes, especially for situation D, were higher. In general, scenario D was difficult for all the tests, however the Wilcoxon-rank-sum test kept the Type II error at a reasonable level for sample sizes of $n=m=50$ and higher. In setting E the Wilcoxon-rank-sum test performed slightly better than the Kolmogorov–Smirnov test.

Settings F and G correspond to scenarios where both, expectation and variance, were equal, which makes a detection of differences between the group rather hard. Again the Wilcoxon-rank-sum test failed to detect the differences, even for sample sizes of $n=m=100$. In both settings the small sample size performance of the NO-Test was better than the one of the Kolmogorov–Smirnov test. On the other hand, especially in setting F the Kolmogorov–Smirnov test obtained lower Type II error rates for high sample sizes.

### Robustness

Finally we investigate how the tests deal with outliers. In this scenario we had sample sizes of $n=m=50$ and successively added up to 15 outliers. Both original data sets were drawn from the same distribution, namely $F=G=N(0,1)$ with the outliers coming from a *N*(0, 10) distribution and were added to the second data set. For all settings 1000 repetitions were performed.

It is desired that tests are robust, such that single outliers don’t effect the test results. However if several data points stray from the data set they might not be outliers any more and a robust test should still pick up on this.

**Table 4:** Influence of outliers on the Type I error for sample size $n=m=50$ for the (niche) overlap test (NO), the Kolmogorov–Smirnov test (KS), and the Wilcoxon-rank-sum test (WR)

	0	1	2	3	4	5	6	7	8	9	10	15
NO	4.2	3.8	5.3	4.2	6.9	6.6	9.7	11.9	14.7	17.8	20.6	40.2
KS	4.8	5.9	5.1	4.7	5.9	6.8	5.0	4.9	6.3	6.9	6.9	7.9
WR	6.7	5.4	5.0	5.0	5.6	6.3	5.0	4.5	4.7	5.8	5.8	4.8

With only a few outliers all three tests stay at the nominal level as it would be desired. With the increase of number of outliers the new test method is the first to start rejecting the null hypothesis when roughly $10\%$ of the sample size are added as outliers. The Wilcoxon-rank-sum test seems rather ignorant against the outliers as even when more than $20\%$ of the sample consists of outliers they stick with the nominal level. For the Kolmogorov–Smirnov test a slight inflation of rejection rate can be observed however it is hardly noticeable.

### Additional simulations

Additional supporting information may be found online in the Supporting Information section at the end of the article. This includes Web Figure 1, referenced in Sect. 3, and Web Appendix A, which shows very small and unequal sample size behavior.

### Data example

To apply the new test procedure to a real life data set we chose an epilepsy treatment data set. The here considered data comes from clinical records of people prescribed perampanel in routine practice. While Rohracher et al. (2018) pooled observational data across Europe, we will only analyze the subset of data collected from Department of Neurology, Christian Doppler Medical Centre, Paracelsus Medical University, Salzburg, Austria. Most of that data was already used in Rohracher et al. (2016) where the study design and data sources are described.

The data set was split into two groups. One containing the people who were still on perampanel at the 12-month follow up, the other containing those that discontinued due to different reasons (e.g., adverse events). The considered variable was the duration of epilepsy, in years, before perampanel initiation. Of the 98 patients in the perampanel group there were 8 missing observations, leaving 90 observations for the analysis. In the second group only one observation of 65 was missing.

The null hypothesis was that there is no difference in duration of epilepsy between the two groups. All three tests do not reject the null hypothesis. The p-values of the new test procedure was equal to 0.908 after application of the Bonferroni-Holmes procedure. The two individual p-values were given by 0.454 and 0.645. The Kolmogorov–Smirnov test had a p-value of 0.211 and the Wilcoxon-rank-sum test had one of 0.350. This implies that no significant difference between the two groups exist.

Those findings agree with the original findings of Rohracher et al. (2018). The higher p-value of the new test procedure compared to the other p-values goes along with the findings of the simulation results.

## Discussion

In this paper, we have introduced a new test statistic for testing equality of distributions based on the concept of overlap. The newly introduced testing method showed overall good behavior in the simulations. Comparing it with standard methods, it showed some advantages.

The presented test procedure is easy to understand and interpret, and fast to calculate. Its wide application area together with its straightforward interpretation could make it a useful alternative to existing tests in medicine and several other disciplines. Even though its performance could be potentially improved via continuity correction it would come at the cost of its comprehensibility. Additional simulations would be necessary to determine if a continuity correction would bring a significant improvement of the test procedure.

The possibilities presented through the idea of the new introduced test procedure are plentiful. One option would be to provide a goodness of fit test in one sample problem. As there the ranks could not be calculated due to the lack of a second sample set another option would need to be found to estimate $I_1$ and $I_2$ as well as the variance estimator. One possibility would be to draw several sample sets from the fixed distribution and use those for estimation of the ranks, like a bootstrap procedure. However this option as well as other approaches should be considered and compared in simulations to find a well suited procedure for the one-sample case.

## Supplementary Information

Below is the link to the electronic supplementary material.

Supplementary material 1 (pdf 301 KB)
