# Supplementary material for: Testing for equality of distributions using the concept of (niche) overlap
Source: Stat Pap (Berl). 2021 May 26;63(1):225–42. doi: 10.1007/s00362-021-01239-y (PMC8801415; doi:10.1007/s00362-021-01239-y)
Supplement: Supplementary file 1 — Supplementary material 1 (pdf 301 KB) [file 362_2021_1239_MOESM1_ESM.pdf]

Statistical Papers manuscript No.
(will be inserted by the editor)

Supplementary Material for ’Testing for Equality of Distributions using the Concept of (Niche) Overlap’

Judith H. Parkinson-Schwarz · Arne C. Bathke

Received: date / Accepted: date

J. H. Parkinson-Schwarz
Department of Mathematics, University of Salzburg, Hellbrunner Straße 34, 5020 Salzburg, Austria
E-mail: judith.parkinson@t-online.de

A. C. Bathke
Department of Mathematics, University of Salzburg, Hellbrunner Straße 34, 5020 Salzburg, Austria

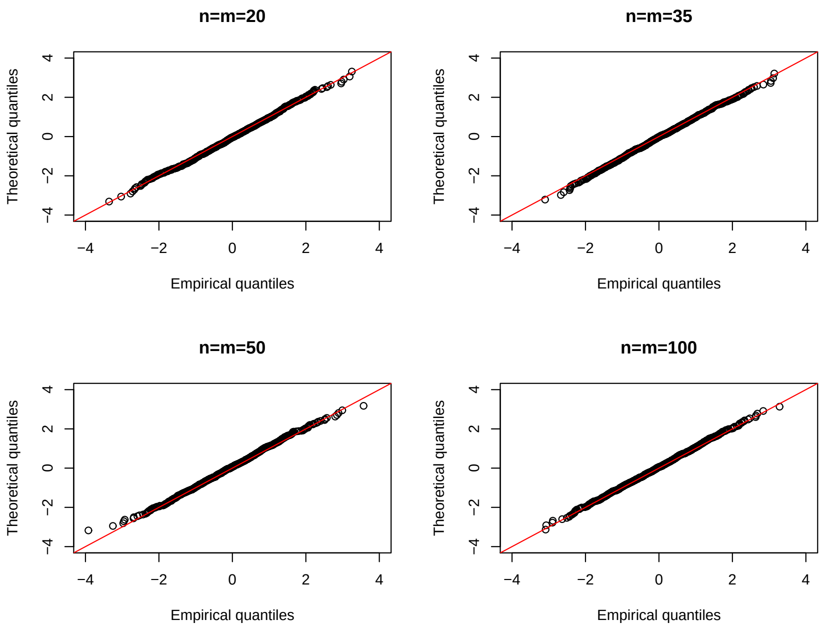

Fig. 1 Q-Q plot of the empirical quantiles of NO_2 (x-axis) versus the theoretical t_{n+m-1} percentiles (y-axis) for different sample sizes corresponding to the theoretical result in the main paper. The red line is the bisecting line.

Simulations

In order to investigate the small sample properties of the test statistics concerning Type I and Type II error, we have performed simulation studies in R (R version 3.2.3, R Core Team, 2017). Additionally, simulations to check the correctness and robustness of the new test procedure were run. The results here are supplementary material for the paper Testing for Equal Distribution using Niche Overlap.

Web Figure 1

In this part we will confirm the limiting distribution as derived in the main paper. Consider data distributed with $F = G = exp(0.5)$. The sample sizes in all 4 settings are equal and given by $20, 35, 50, 100$. For each setting we ran 1000 simulations.

The Q-Q plots of the empirical quantiles vs. the t-distribution percentiles can be seen in Figure 1. The results are similar to those reported in the paper.

Web Appendix A

In this part we focus on the influence of very small and unbalanced sample size settings on the performances. The combinations of the sample sizes (n,m) are: $(5,5)$, $(5,10)$, $(10,10)$, $(10,20)$, $(10,50)$, $(25,100)$. For all combinations of sample sizes we simulated two settings where the null hypothesis holds true, $N1 : F = G = N(0,1)$ and $N2 : F = G = Exp(1/2)$, and five settings where the null hypothesis was not correct. The alternative settings were $A1 : F = N(0,1), G = N(1.5,1)$, $A2 : F = N(0,1), G = \mathscr{X}_2^2$, $A3 : F = U(0,1), G = B(2,4)$, $A4 : F = Exp(2), G = B(2,4)$, and $A5 : F = B(2,4), G = Exp(2)$.

Table 1 Error rates for different unbalanced sample sizes for two nulls and six alternatives.

	(5,5)	(5,10)	(10,10)	(10,20)	(10,50)	(25,100)
NO						
N1	10.9	12.5	3.9	6.6	7.7	3.8
N2	11.7	11.7	5.1	5.5	9.4	5.8
A1	39.5	33.3	23.7	11.7	9.5	0.5
A2	34.4	35.5	24.9	16.1	10.1	0.7
A3	77.5	78.2	70.6	68.9	65.4	20.4
A4	86.7	85.5	76.4	75.9	74.9	30.5
KS						
N1	1.2	2.7	1.2	2.8	3.9	4.2
N2	0.8	2.0	1.4	2.3	3.5	4.8
A1	82.0	56.3	43.4	15.1	5.5	0.0
A2	86.3	61.3	52.2	22.3	8.2	0.0
A3	94.9	87.2	89.5	71.1	56.1	18.3
A4	99.1	93.4	97.0	90.3	78.7	56.3
WR						
N1	3.5	4.6	4.0	5.2	3.9	4.6
N2	3.1	4.3	4.6	4.2	3.8	5.4
A1	57.7	36.0	14.5	4.9	1.8	0.0
A2	61.0	36.3	16.9	6.8	1.8	0.0
A3	88.8	81.1	77.2	65.5	59.3	26.8
A4	96.3	92.0	93.0	89.4	85.5	84.2

The error rates of the three tests can be found in Table 1. Considering the two null hypothesis the similar behaviour can be noticed as in the balanced sample size setting. The niche-overlap test proves to be too liberal for very small sample sizes while the Kolmogorov-Smirnov test seems to be too conservative. In almost all settings of the alternatives our new test outperforms both of the others, in some settings even significantly. Yet, especially for sample sizes $(5,5)$ and $(5,10)$ one should keep in mind how liberal the niche-overlap test is. Which consequently leads to a lower Type II error for those sample sizes. The higher sample sizes show that the niche-overlap test performs better in those settings even when the Type I error is close to the normal level, implying that the low Type II error for very small sample sizes is only partly explained by the liberal behaviour of the test.
